# Correlates of knowledge of genetic diseases and congenital anomalies among pregnant women attending antenatal clinics in Lagos, South-West Nigeria

**DOI:** 10.11604/pamj.2021.38.310.26636

**Published:** 2021-03-28

**Authors:** Chibuzor Franklin Ogamba, Alero Ann Roberts, Ochuwa Adiketu Babah, Chibuikem Anthony Ikwuegbuenyi, Oluwaseun Joseph Ologunja, Oluyinka Kehinde Amodeni

**Affiliations:** 1 Faculty of Clinical Sciences, College of Medicine, University of Lagos, Lagos, Nigeria,; 2 Department of Community Health and Primary Care, College of Medicine, University of Lagos, Lagos, Nigeria,; 3 Department of Obstetrics and Gynaecology, College of Medicine, University of Lagos/Lagos University Teaching Hospital, Surulere, Lagos, Nigeria

**Keywords:** Genetic diseases, congenital anomalies, pregnant women, prenatal diagnosis

## Abstract

**Introduction:**

genetic diseases and congenital anomalies place a significant burden on the health of new-borns and their mothers. Despite the availability of a variety of prenatal screening tests, mothers' knowledge has been documented to determine uptake. This study aims to assess the knowledge of pregnant women about birth defects and the associated correlates with regard to willingness to do prenatal screening.

**Methods:**

a cross-sectional descriptive study was conducted among 422 antenatal mothers recruited sequentially as they attended antenatal clinics at the Lagos University Teaching Hospital. An interviewer-administered questionnaire was used to determine their knowledge of birth defects and willingness to do prenatal testing.

**Results:**

majority of the participants (92.2%) had at least secondary education. The mean total knowledge score of the respondents was 63%. Age and knowledge scores were not significantly correlated (r=-0.071, p=0.14). Being employed predicted higher knowledge scores (95% CI: 0.09, 2.09, p=0.03). Respondents who had primary school education and those who replied “I don't know” to willingness to test had significantly lower knowledge scores (95% CI: -15.01, -1.19, p=0.02 and 95% CI: -4.52, -0.68, p=0.01 respectively). Majority (79.1%) of the respondents were willing to undergo testing. Respondents' level of education was significantly associated with willingness to test (p=0.03).

**Conclusion:**

the observed knowledge gaps were considerable. There is need for improvement in education, the empowerment of women and access to quality healthcare including prenatal screening.

## Introduction

Genetic diseases and congenital anomalies are a recognised public health problem because of the huge morbidity and mortality rates associated with them [1-4]. The deaths of about 303,000 neonates each year worldwide have been attributed to birth defects and about 7.9 million children are born with serious birth defects [1]. The children who survive go on to face higher chances of physical, cognitive and social disabilities all through life which place huge financial, emotional, psychological and social burdens on their families. Research has documented that birth defects are responsible for miscarriages, pre-term deliveries and stillbirths and therefore constitute a significant drawback to the achievement of global health goals [5-7]. Most birth defects for which causes can be determined are as a result of chromosomal anomalies and single gene mutations with environmental factors accounting for the rest. However, this only accounts for less than a quarter of birth defects. For the greater majority, the causes are largely unknown [8-10].

Low- and middle-income countries have been reported to have the highest prevalence and burden of serious birth defects with an attending 95% mortality rates among affected children [1,3]. Several studies have reported a range of prevalence of birth defects from 0.5% prevalence of orofacial clefts and 0.7% prevalence of cervical ribs to 6.9% prevalence of external congenital birth defects in South-West Nigeria [2,11-18]. In addition to factors such as the socio-economic conditions of people living in low and middle income countries, a lack of knowledge and education of mothers on genetic diseases and congenital anomalies have been highlighted as major factors which contribute to the huge disparities in incidence between developing and developed countries which do not appear to have undergone much change historically till date [19-23].

Certain genetic diseases and birth defects are preventable especially through interventions such as folic acid supplementation before pregnancy [24], iodination of food items [25,26], immunization with rubella vaccine [27], improvement of nutritional status among mothers [28], premarital genotype screening [29], as well as prompt diagnosis and treatment of pre-existing health conditions before pregnancy, it takes an educated mother to know all these and to know how and when to seek help from a health facility [30].

Research has reported that in most developing countries, a high percentage of women above 35 years of age give birth without any available community education, medical genetic screening and diagnostic services as well as proper antenatal care [3,31]. Adequate knowledge has been shown to lead to the prevention of genetic diseases and congenital anomalies and also prevent physical, economic and social consequences associated with birth defects and lead to increased chances of a better quality of life for both mother and child [32-34]. The role adequate knowledge of genetic diseases and birth defects among prospective mothers plays in the reduction of genetic diseases and congenital anomalies underpins the objectives of this study to investigate the level of knowledge of genetic diseases and congenital anomalies and examine the willingness to undergo testing among pregnant women attending antenatal clinics at the Lagos University Teaching Hospital, Idi-Araba as well as socio-demographic correlates of knowledge and willingness.

## Methods

**Sample size and selection:** this was a hospital-based cross-sectional descriptive study carried out at the Lagos University Teaching Hospital (LUTH), Idi-Araba, Lagos which is one of only two tertiary centers in the state and the only one run by the Federal Government and thus serves as a referral center for obstetrics and gynecology cases in the region. Lagos State is located in the South-Western part of Nigeria. This study was conducted on 422 pregnant women who attended antenatal clinics at LUTH between February 2018 and October 2019. Consenting pregnant women were recruited as they presented to the facility for antenatal clinics using sequential sampling technique based on the eligibility criteria, until the sample size was achieved. Pregnant women who were not previously registered and all those who refused informed consent were excluded from the study. The sample size was calculated using the Cochran formula, with a prevalence value of 50% based on previous research [35], significance level of 95% and an alpha level of 0.05, allowing an attrition value of 10% for contingencies such as non-response or recording error. This research was part of a larger study and analysis of data was done as soon as sample size was achieved.

**Data collection instrument:** the main outcome variable was the knowledge respondents had about birth defects. Secondary outcome examined was the proportion of women who had or would undertake a prenatal test to screen for genetic diseases or congenital anomalies. This was examined by the response given to the question of willingness to test among respondents. An interviewer-administered questionnaire adapted from previous studies [1,3] was used to collect data and included both closed and open-ended questions divided into three sections. The questionnaire was pretested before use on women attending antenatal clinic at another health facility. This was used to make adjustments as well as to improve the comprehensibility and local context of the tool. Face validity of the questionnaire was evaluated by a consultant obstetrician and gynaecologist and a consultant public health physician. The questionnaire started with questions ascertaining the socio-demographic characteristics of respondents including age (at last birthday, recorded in years), marital status (recorded as single, married and separated/divorced), religion (recorded as 'Islam', 'Christianity', 'traditional' and 'others'), highest level of education, employment status, parity and the number of antenatal clinics attended.

Respondents' knowledge of genetic diseases and congenital anomalies was examined using 26 knowledge questions that sought knowledge of the nature of genes, causes of genetic diseases and birth defects, risk factors and prevention. Sickle cell disease and Down syndrome were used to assess for knowledge of genetic diseases as these are the most common in our environment. Also included was an item for respondents to indicate their major source of knowledge. Correct responses were scored 1 and incorrect and 'don't know' responses were scored 0. 'Good' knowledge was determined if the respondent scored 14 and above, scores of 7-13 was determined to be 'fair' while scores of 6 and under was determined to be 'poor' knowledge. There was a question that examined whether respondents had previously taken a prenatal test, either in this pregnancy or a previous one. Respondents were then asked about their willingness to undergo prenatal testing.

**Ethics:** ethical approval for the study was obtained from the Health Research and Ethics Committee of LUTH (ADM/DCST/HREC/APP/1970). Informed consent was obtained from the participants after an information session by indicating their responses in the boxes provided on the tool.

**Data analysis:** data was entered and coded using Microsoft Excel 2007 and analysed using SPSS version 20. Descriptive statistics were used to describe the socio-demographic characteristics and the results were presented as means, frequencies and percentages. Spearman´s rank correlation was used to test for correlation between continuous variables of age and knowledge scores. Chi-square tests were used to test for associations between socio-demographic variables and willingness to test, and statistical significance was determined at p<0.05.

## Results

**Socio-demographic characteristics of the participants:** the mean age ± S.D. of respondents was 32.5 ± 5.3 years, with majority (242/422, 57.3%) within the age range 31 - 40 years. They were predominantly Christian (359/422, 85.1%), married (408/422, 96.7%), employed (321/422, 76.1%) and attained at least secondary school level of education (389/422, 92.2%). Majority of the respondents (255/422, 60.4%) were multigravidas with a range of 2-4 pregnancies and most of the respondents (248/422, 58.8%) have made less than 10 antenatal clinic visits. Table 1 summarizes the socio-demographic characteristics of the respondents.

**Table 1:** socio-demographic characteristics of respondents

Characteristic	Mean ± SD [Range]	Frequency (%) (N=422)
Age	32.5 ± 5.3 [18 - 69]	-
Knowledge score	12.6 ± 4.6 [0 - 20]	-
**Education level**		
None	-	7 (1.7)
Primary	-	2 (0.5)
Secondary and above	-	389 (92.2)
Vocational	-	24 (5.7)
**Employment status**		
Employed	-	321 (76.1)
Unemployed	-	101 (23.9)
**Marital status**		
Single	-	10 (2.4)
Married	-	408 (96.7)
Separated/widowed	-	4 (0.9)
**Gravidity**		
Primigravida	-	137 (32.5)
2 - 4	-	255 (60.4)
More than 4	-	30 (7.1)
**Religion**		
Christian	-	359 (85.1)
Islam	-	58 (13.7)
Traditional/other	-	5 (1.2)
Number of antenatal visits		
<10	-	248 (58.8)
From 10 -19	-	113 (26.8)
From 20-29	-	39 (9.2)
≥30	-	22 (5.2)

**Knowledge of genetic diseases and congenital anomalies:** majority of the respondents (285/422, 67.5%) knew that genes come in pairs, one copy from each parent, (219/422, 51.9%) knew that genetic diseases are not caused by an infection, (234/422, 55.5%) knew that inheriting two abnormal genes from both parents would result in a genetic disease, (329/422, 78.0%) knew that genetic diseases can affect a baby developing in the womb, (266/422, 63.0%) knew that sickle cell disease can be inherited, (321/422, 76.1%) knew that one family member having a genetic disease does not mean all family members would have the disease, (286/422, 67.8%) knew that birth defects can be acquired by a baby developing in the womb, (305/422, 72.3%) were aware that most birth defects are preventable, (332/422, 78.7%) knew that birth defects can be managed medically and (231/422, 54.7%) that neural tube defects represented a pathology in the baby´s brain or spinal cord. Just half of the respondents (211/422, 50.0%) knew that it is possible to have the gene for a genetic disease and not have symptoms as a result. However, majority (244/422, 57.8%) did not know that Down syndrome is a genetic disease and (291/422, 69%) did not know that birth defects were not acquired by pregnant women. Table 2 summarizes these findings.

**Table 2:** respondents' knowledge of genetic diseases and congenital anomalies

Characteristics	Yes (%)	No (%)	Don't know (%)
**Knowledge of genetic diseases and birth defects**			
Genes come in pairs, one copy from each parent	285 (67.5)	21 (5.0)	116 (27.5)
Genetic diseases are diseases that are caused by inheriting two abnormal genes from the parent	234 (55.5)	60 (14.2)	128 (30.2)
Genetic diseases are caused by an infection	58 (13.7)	219 (51.9)	145 (34.4)
Genetic diseases can affect a baby developing in the womb	329 (78.0)	18 (4.3)	75 (17.8)
All family members develop the disease, if one family member has a genetic disease	22 (5.2)	321 (76.1)	79 (18.7)
Sickle cell disease can be inherited	266 (63.0)	108 (25.6)	48 (11.4)
Marriage of two AS partners could lead to a child being born with sickle cell disease	391 (92.7)	8 (1.9)	23 (5.5)
Down syndrome is a genetic disease	178 (42.2)	73 (17.3)	171 (40.5)
There is no risk of Down syndrome in my children, if I have no family member with Down syndrome	129 (30.6)	128 (30.3)	165 (39.1)
It is possible to have a gene for a genetic disease but not have symptoms of the disease	211 (50.0)	47 (11.1)	164 (38.9)
Birth defects are exclusively acquired by pregnant women	134 (31.8)	131 (31.0)	157 (37.2)
Birth defects can be acquired by baby developing in the womb	286 (67.8)	34 (8.1)	102 (24.2)
Most birth defects are preventable	305 (72.3)	27 (6.4)	90 (21.3)
Birth defects can be managed medically	332 (78.7)	10 (2.4)	80 (19.0)
Alcohol consumption during pregnancy increases the risk of having a child with birth defects	338 (80.1)	14 (3.3)	70 (16.6)
Using un-prescribed drugs will increase the risk of having a child with birth defects	370 (87.7)	4 (0.9)	48 (11.4)
Smoking before and during pregnancy will increased the risk of having a child with birth defects	365 (86.5)	7 (1.7)	50 (11.8)
Advanced maternal age increases the risk of having a child with genetic diseases and birth defects	189 (44.8)	82 (19.4)	151 (35.8)
Women who are not vaccinated against rubella and who have not taken folic acid before pregnancy are at increased risk of having a child with birth defects	217 (51.4)	57 (13.5)	148 (35.1)
A neural tube defect is when something is wrong with the baby's brain or spinal cord	231 (54.7)	8 (1.9)	183 (43.3)

**Knowledge of the risk and prevention of genetic diseases and congenital anomalies:** majority of the respondents (391/422, 92.7%) knew that marriage of two people carrying the AS genotype could lead to a child with sickle cell disease and that alcohol consumption 338 (80.1%), using un-prescribed drugs 370 (87.7%) and smoking during pregnancy 365 (86.5%) increased the risk of having children with birth defects. However, only 128 (30.3%) knew that there could be a risk of Down syndrome in their children if no family member of theirs has Down syndrome, 233 (55.2%) did not know that advanced maternal age increases the risk of having a child with genetic diseases and birth defects and only 217 (51.4%) knew that women who are not vaccinated against rubella and who have not taken folic acid before pregnancy are at increased risk of having a child with birth defects (Table 2). Overall, barely half of the respondents 209 (49.5%) had good knowledge scores (14-20) while 165 (39.1%) had fair knowledge scores (7-13). However, mean knowledge score ± S.D. of the population was 12.6 ± 4.6. Respondents listed health personnel 149 (35.3%), the internet 125 (29.6%) and the media 101 (23.9%) as their major sources of knowledge of genetic diseases and congenital anomalies.

**Correlates of knowledge and willingness to test:** the correlates of respondents´ level of knowledge were subjected to bivariate and regression analyses with their socio-demographic characteristics. Respondents who were employed had significantly higher knowledge scores than those who were not (95% CI: 0.09, 2.09, p=0.03). Those who had primary school level of education and those who replied ‘I don´t know´ to willingness to test had significantly lower knowledge scores (95% CI: -15.01, -1.19, p=0.02 and 95% CI: -4.52, -0.68, p=0.01 respectively). Respondents´ age was not significantly correlated with knowledge scores (r=-0.071, p=0.14). There were no significant relationships between respondents´ level of knowledge and other socio-demographic variables (Table 3). Majority of the respondents (334/422, 79.1%) were willing to undergo testing. Only education was found to be significantly associated with willingness to test, p=0.03 (Table 4).

**Table 3:** socio-demographic factors as joint predictors of knowledge score

Variables	Change in knowledge score (95% CI)	P-value
Age	-0.02 (-0.12, 0.07)	0.62
**Education**		
Primary	-8.10 (-15.01, -1.19)	0.02
Secondary and above	0.60 (-2.77, 3.96)	0.73
Vocational	-1.19 (-4.98, 2.60)	0.54
**Employment**		
Unemployed	Ref	-
Employed	1.09 (0.09, 2.09)	0.03
**Marital status**		
Married	Ref	-
Single	-2.61 (-5.36, 0.15)	0.06
Separated/widowed	0.22 (-4.23, 4.66)	0.92
**Gravidity**		
1	Ref	-
2	-0.51 (-1.56, 0.53)	0.34
3	-0.61 (-1.90, 0.68)	0.36
4	-0.74 (-2.24, 0.76)	0.33
>4	-0.51 (-2.36, 1.35)	0.59
**Religion**		
Christian	Ref	-
Islam	-0.50 (-1.71, 0.70)	0.41
Other	-3.77 (-7.61, 0.06)	0.05
**Number of antenatal visits**		
<10	Ref	
10-19	0.42 (-1.17, 2.35)	0.40
20-29	0.98 (-0.51. 2.47)	0.20
≥30	0.74 (-1.22, 2.71)	0.46
**Willingness to test**		
No	Ref	-
Don't know	-2.60 (-4.52, -0.68)	0.01
Yes	1.28 (-0.31, 2.86)	0.11

**Table 4:** chi-squared tests of socio-demographic characteristics with willingness to test

Variable	Chi-squared statistic	Degrees of freedom	P-value
Age	4.49	9	0.85
Education	14.69	9	0.03
Employment	4.82	3	0.19
Marital Status	2.11	6	0.94
Gravidity	11.09	12	0.46
Religion	3.44	6	0.64
Number of antenatal visits	8.03	9	0.49

## Discussion

Knowledge of genetic diseases and congenital anomalies and how they can be prevented is important to reducing the prevalence of morbidity that puts pressure on the health system. This study showed that barely half of the respondents had good knowledge overall of the causes and consequences of genetic diseases and congenital anomalies. However, this was higher than what was reported by another study where only a quarter of the mothers reported awareness of birth defects [31]. The overall paucity of knowledge was despite the fact that most of the respondents had attained at least secondary education, although there was no significant association between their level of education and the overall knowledge of genetic diseases and birth defects. This finding agreed with a study done in Ghana but did not agree with other studies done in Nigeria and Iran [31,32,36]. The majority of the study participants were in the age range 31-40 years similar to other studies in South-Western Nigeria and among the age range that is at higher risk of having children with a congenital anomaly [35,37,38]. However, unlike an earlier study done in Nigeria, where age over 30 years was associated with better knowledge, the median age of participants in our study did not show a statistically significant association with knowledge [31]. This finding thus suggests that knowledge is not age dependent as far as health education is concerned but more likely related to interest in seeking for knowledge.

The results from this study proves an opportunity for establishing a practice of improving early detection of genetic disease and congenital anomalies by offering health education to all pregnant women early in pregnancy or preferably in the preconception period in this regard. This will be dependent on mothers having access to scientifically accurate sources of information. The respondents in this study reported that they had mostly obtained information from healthcare workers and hospital staff. The source of information being healthcare workers was also reported by several studies that demonstrated the importance of mothers´ knowledge and childhood survival [39-43]. The pertinence of this is the link to attendance at antenatal clinics. The World Health Organization antenatal clinic model now recommends a minimum of eight antenatal clinic contacts to be made by a pregnant woman in each pregnancy for a positive pregnancy experience [44]. The gaps in knowledge demonstrated in this study are particularly relevant in that there has been a lot done to create awareness of sickle cell disease and how to prevent it through premarital genetic testing. Previous studies have linked the paucity of knowledge and uptake of services to non-availability of prenatal testing services and religious persuasions [45,46]. This also carries into the low levels of knowledge of neural tube defects (NTDs) despite the universal use of prenatal folate supplementation. Maternal pre-conceptual folic acid supplementation has been highly correlated with the prevention of neural tube defects as the neural tube forms by the 28^th^ day of gestation at which time the mother does not know that she is pregnant. Increased awareness about the need for pre-conceptual folic acid supplementation among women of child-bearing age is therefore needed [1].

The key role of physicians in the education of pregnant women about birth defects emphasizes the need for increased communication between physicians and patients. A good number of the respondents did not know that advanced maternal age, lack of vaccination against rubella and pre-conceptual folic acid supplementation were risk factors for having children with congenital anomalies. Respondents are not properly informed about NTDs and this likely has an effect on the willingness of respondents to undergo prenatal screening [31,47,48].

Knowledge of Down syndrome was also found to be low in this study despite the fact that its prevalence in society matches prevalence anywhere in the world. Though the prevalence of congenital anomalies especially Down syndrome has not been properly investigated and recorded, the earliest study done on the incidence of Down syndrome in Nigeria found 1 in 865 live births to be affected by Down syndrome [49]. Also, a study done in the South-South region of the country found Down syndrome to constitute 3.3% of the 452 births with congenital malformations recorded [50]. The added difficulties of coping with children born with genetic diseases and congenital anomalies make it imperative that more comprehensive data collection and testing be instituted, more awareness needs to be created for the possibility of reducing the prevalence [51-53].

Correlates of knowledge reported in this study included being employed which was a predictor of better knowledge scores. This corresponds to the findings of a similar study where participants who were of the working class especially the upper/middle social classes were more likely to be aware about birth defects [31], thus lending credence to the idea that those of the working class have better opportunities to access avenues of information than those who are unemployed. Majority of the respondents, over three-quarters of the population were willing to undergo testing. In our study significantly lower knowledge scores were observed among pregnant women who were unsure about willingness to test. This suggests that knowledge of genetic diseases and congenital anomalies may play a role in informed choice about prenatal screening as has been previously highlighted [54,55]. Willingness to test was associated with respondents´ level of education and it is important that this is leveraged on so that screening can ethically be included into routine care. This finding agreed with other studies done both in Nigeria and elsewhere where acceptance of prenatal tests was influenced by educational attainment [39,56-59].

## Conclusion

Despite the fact that almost half of the respondents had good knowledge scores, there were significant gaps in knowledge observed and this is sufficient to warrant further study to understand the nuances of effective health education regarding genetic diseases and congenital anomalies. Our findings concur with other research that underscores the need for increased awareness, especially about the effects of advanced maternal age and the importance of both proper vaccinations and pre-conceptual folic acid supplementation and indeed about genetic diseases and congenital anomalies in general. We recommend increasing awareness about the need for antenatal clinic contacts and improving the contents of antenatal health education sessions to include topics about genetic diseases and birth defects and ways to prevent them. We also recommend improved women education and empowerment which ensures that every mother has access to healthcare.

**Data availability statement:** the datasets generated during and/or analysed during the current study are available from the corresponding author on reasonable request.

### What is known about this topic

- Birth defects are a recognized public health problem responsible for complications associated with maternal and child health;
- Some genetic diseases and congenital anomalies are preventable via a couple of interventions, knowledge of which is a prerequisite for pregnant women or intending mothers;
- Low and middle income countries like Nigeria have been reported to have a significant burden of genetic diseases and congenital anomalies associated with problems such as poor socioeconomic conditions as well as a lack of knowledge and education of mothers.

### What this study adds

- There are significant knowledge gaps concerning genetic diseases and birth defects especially about the effects of advanced maternal age and the importance of both proper vaccinations and pre-conceptual folic acid supplementation in Lagos;
- Being employed is a predictor of better knowledge of genetic diseases and congenital anomalies and their prevention in Lagos;
- Poor educational status is a predictor of poor knowledge about genetic diseases and congenital anomalies and education is associated with willingness to test in Lagos; poor knowledge of genetic diseases and birth defects is associated with indecision about testing.

## References

[1] De Silva J, Amarasena S, Jayaratne K, Perera B (2019). Correlates of knowledge on birth defects and associated factors among antenatal mothers in Galle, Sri Lanka: a cross-sectional analytical study. BMC Pregnancy Childbirth.

[2] Ajao AE, Adeoye IA (2019). Prevalence, risk factors and outcome of congenital anomalies among neonatal admissions in OGBOMOSO, Nigeria. BMC Pediatr.

[3] Bello AI, Acquah AA, Quartey JN, Hughton A (2013). Knowledge of pregnant women about birth defects. BMC Pregnancy Childbirth.

[4] Peter AI, Ekong MB, Ekanem TB, Idonrenyin UU, Edagha II, Davies KG (2013). Attitude and knowledge of pregnant women attending antenatal clinic at St Lukes hospital, Anua in Uyo, Nigeria towards congenital anomalies. Ibom Med J.

[5] Fawole AO, Shah A, Tongo O, Dara K, El-Ladan AM, Umezulike AC (2011). Determinants of perinatal mortality in Nigeria. Int J Gynaecol Obstet.

[6] Swanson JR, Sinkin RA (2013). Early births and congenital birth defects: a complex interaction. Clin Perinatol.

[7] Roncancio CP, Misnaza SP, Peña IC, Prieto FE, Cannon MJ, Valencia D (2018). Trends and characteristics of fetal and neonatal mortality due to congenital anomalies, Colombia 1999-2008. J Matern-Fetal Neonatal Med.

[8] Institute of Medicine (US) Committee on Improving Birth Outcomes (2003). Reducing birth defects: meeting the challenge in the developing world.

[9] Lancaster PAL (2011). Causes of birth defects: lessons from history. Congenit Anom (Kyoto).

[10] Feldkamp ML, Carey JC, Byrne JLB, Krikov S, Botto LD (2017). Etiology and clinical presentation of birth defects: population-based study. BMJ.

[11] Bakare TIB, Sowande OA, Adejuyigbe OO, Chinda JY, Usang UE (2009). Epidemiology of external birth defects in neonates in Southwestern Nigeria. Afr J Paediatr Surg.

[12] Wieacker P, Steinhard J (2010). The prenatal diagnosis of genetic diseases. Dtsch Ärztebl Int.

[13] Obu HA, Chinawa JM, Uleanya ND, Adimora GN, Obi IE (2012). Congenital malformations among newborns admitted in the neonatal unit of a tertiary hospital in Enugu, South-East Nigeria - a retrospective study. BMC Res Notes.

[14] Butali A, Adeyemo WL, Mossey PA, Olasoji HO, Onah II, Adebola A (2014). Prevalence of orofacial clefts in Nigeria. Cleft Palate Craniofac J.

[15] Ezeofor SN, Njeze NR, Aghaji MN, Onuh AC, Obikili EN (2016). The prevalence of cervical ribs in Enugu, Nigeria. Niger J Clin Pract.

[16] Offor I, Awodele O, Oshikoya KA (2019). Drug-related teratogenic and pathologic causes of birth defects in a tertiary hospital in Southwestern Nigeria. Pharmacol Res Perspect.

[17] Abbey M, Oloyede OA, Bassey G, Kejeh BM, Otaigbe BE, Opara PI (2017). Prevalence and pattern of birth defects in a tertiary health facility in the Niger Delta area of Nigeria. Int J Womens Health.

[18] Adeyemo AA, Gbadegesin RA, Omotade OO (1997). Major congenital malformations among neonatal referrals to a Nigerian university hospital. East Afr Med J.

[19] Ojofeitimi EO, Elegbe I (1984). Mothers' beliefs as to causation and prevention of birth defects in Ile-Ife, Nigeria. J R Soc Health.

[20] Olasoji HO, Ugboko VI, Arotiba GT (2007). Cultural and religious components in Nigerian parents' perceptions of the aetiology of cleft lip and palate: implications for treatment and rehabilitation. Br J Oral Maxillofac Surg.

[21] Fransen MP, Essink-Bot M-L, Oenema A, Mackenbach JP, Steegers EAP, Wildschut HIJ (2007). Ethnic differences in determinants of participation and non-participation in prenatal screening for Down syndrome: a theoretical framework. Prenat Diagn.

[22] Crombag NM, Bensing JM, Iedema-Kuiper R, Schielen PC, Visser GH (2013). Determinants affecting pregnant women's utilization of prenatal screening for Down syndrome: a review of the literature. J Matern Fetal Neonatal Med.

[23] Michael AI, Jarrett OO (2019). Parental views on plastic surgery for Down syndrome: an African perspective. Pan African Medical Journal.

[24] De-Regil LM, Peña-Rosas JP, Fernández-Gaxiola AC, Rayco-Solon P (2015). Effects and safety of periconceptional oral folate supplementation for preventing birth defects. Cochrane Database Syst Rev.

[25] Obican SG, Jahnke GD, Soldin OP, Scialli AR (2012). Teratology public affairs committee position paper: iodine deficiency in pregnancy. Birt Defects Res A Clin Mol Teratol.

[26] Zimmermann MB (2012). The effects of iodine deficiency in pregnancy and infancy. Paediatr Perinat Epidemiol.

[27] Lambert N, Strebel P, Orenstein W, Icenogle J, Poland GA (2015). Rubella. Lancet.

[28] Abu-Saad K, Fraser D (2010). Maternal nutrition and birth outcomes. Epidemiol Rev.

[29] Burke W, Tarini B, Press NA, Evans JP (2011). Genetic screening. Epidemiol Rev.

[30] Tinker SC, Carmichael SL, Anderka M, Browne ML, Caspers Conway KM, Meyer RE (2015). Next steps for birth defects research and prevention: the birth defects study to evaluate pregnancy exposures (BD-STEPS). Birt Defects Res A Clin Mol Teratol.

[31] Lawal TA, Yusuf OB, Fatiregun AA (2015). Knowledge of birth defects among nursing mothers in a developing country. Afr Health Sci.

[32] Owotade FJ, Ogundipe OK, Ugboko VI, Okoje VN, Olasoji HO, Makinde ON (2014). Awareness, knowledge and attitude on cleft lip and palate among antenatal clinic attendees of tertiary hospitals in Nigeria. Niger J Clin Pract.

[33] Ogunlesi TA, Fetuga MB, Adekanmbi AF (2013). Mothers' knowledge about birth asphyxia: the need to do more. Niger J Clin Pract.

[34] Egube BA, Ofili AN, Isara AR, Onakewhor JU (2013). Neonatal jaundice and its management: knowledge, attitude, and practice among expectant mothers attending antenatal clinic at University of Benin Teaching Hospital, Benin City, Nigeria. Niger J Clin Pract.

[35] Adekanbi AOA, Olayemi OO, Fawole AO (2014). The knowledge base and acceptability of prenatal diagnosis by pregnant women in Ibadan. Afr J Reprod Health.

[36] Masoumeh P, Vahid K, Hamid AM, Khosheh K, Samira K (2015). Knowledge of pregnant women about congenital anomalies: a cross-sectional study in north of Iran. Indian J Med Sci.

[37] Ogamba CF, Roberts AA, Balogun MR, Ikwuegbuenyi CA (2018). Genetic diseases and prenatal genetic testing: knowledge gaps, determinants of uptake and termination of pregnancies among antenatal clinic attendees in Lagos, Southwest Nigeria. Ann Med Health Sci Res.

[38] Morris JK, Alberman E (2009). Trends in Down's syndrome live births and antenatal diagnoses in England and Wales from 1989 to 2008: analysis of data from the National Down Syndrome Cytogenetic Register. BMJ.

[39] Al-Ayed IH (2010). Mothers' knowledge of child health matters: are we doing enough. J Fam Community Med.

[40] Merga N, Alemayehu T (2015). Knowledge, perception and management skills of mothers with under-five children about diarrhoeal disease in indigenous and resettlement communities in Assosa District, Western Ethiopia. J Health Popul Nutr.

[41] Mukunya D, Kizito S, Orach T, Ndagire R, Tumwakire E, Rukundo GZ (2014). Knowledge of integrated management of childhood illnesses community and family practices (C-IMCI) and association with child undernutrition in Northern Uganda: a cross-sectional study. BMC Public Health.

[42] Berhea TA, Belachew AB, Abreha GF (2018). Knowledge and practice of essential newborn care among postnatal mothers in Mekelle City, North Ethiopia: a population-based survey. PloS One.

[43] Nigatu SG, Worku AG, Dadi AF (2015). Level of mother's knowledge about neonatal danger signs and associated factors in North West of Ethiopia: a community-based study. BMC Res Notes.

[44] World Health Organization (2016). WHO recommendations on antenatal care for a positive pregnancy experience. Geneva: World Health Organization.

[45] Kagu MB, Abjah UAM, Ahmed SG (2004). Awareness and acceptability of prenatal diagnosis of sickle cell anaemia among health professionals and students in North Eastern Nigeria. Niger J Med.

[46] Famuyiwa OO, Aina OF (2010). Mother's knowledge of sickle-cell anaemia in Nigeria. Int Q Community Health Educ.

[47] Lang CW, Stark AP, Acharya K, Ross LF (2009). Maternal knowledge and attitudes about newborn screening for sickle cell disease and cystic fibrosis. Am J Med Genet A.

[48] Bener A, Al Maadid MGA, Al-Bast DAE, Al-Marri S (2006). Maternal knowledge, attitude and practice on folic acid intake among Arabian Qatari women. Reprod Toxicol.

[49] Adeyokunnu AA (1982). The incidence of Down's syndrome in Nigeria. J Med Genet.

[50] Ekanem TB, Okon DE, Akpantah AO, Mesembe OE, Eluwa MA, Ekong MB (2008). Prevalence of congenital malformations in Cross River and Akwa Ibom states of Nigeria from 1980-2003. Congenit Anom (Kyoto).

[51] Manikandan K, Seshadri S (2017). Down syndrome screening in India: are we there yet. J Obstet Gynaecol India.

[52] Jan YE, Binjahlan MM, Alqurashi AG, Alqurashi GG, Zirari MA, Alturkistani FM (2017). Assessment of knowledge: attitude and practice toward Down syndrome in Jeddah City, Saudi Arabia 2016. Egypt J Hosp Med.

[53] Sijeeni AS (2016). Understanding the experiences of mothers caring for children with Down syndrome in Saudi Arabia. Brisbane, Queensland University of Technology.

[54] Stefansdottir V, Skirton H, Jonasson K, Hardardottir H, Jonsson JJ (2010). Effects of knowledge, education and experience on acceptance of first trimester screening for chromosomal anomalies. Acta Obstet Gynecol Scand.

[55] Etchegary H, Potter B, Howley H, Cappelli M, Coyle D, Graham I (2008). The influence of experiential knowledge on prenatal screening and testing decisions. Genet Test.

[56] Kemper AR, Mahle WT, Martin GR, Cooley WC, Kumar P, Morrow WR (2011). Strategies for implementing screening for critical congenital heart disease. Pediatrics.

[57] Li D-K, Karlberg K, Wi S, Norem C (2008). Factors influencing women's acceptance of prenatal screening tests. Prenat Diagn.

[58] Sahin NH, Gungor I (2008). Congenital anomalies: parents' anxiety and women's concerns before prenatal testing and women's opinions towards the risk factors. J Clin Nurs.

[59] Seavilleklein V (2009). Challenging the rhetoric of choice in prenatal screening. Bioethics.

